# One-Step Preparation of Antifouling Polysulfone Ultrafiltration Membranes via Modification by a Cationic Polyelectrolyte Based on Polyacrylamide

**DOI:** 10.3390/polym12051017

**Published:** 2020-04-30

**Authors:** Tatiana V. Plisko, Alexandr V. Bildyukevich, Katsiaryna S. Burts, Sergey S. Ermakov, Anastasia V. Penkova, Anna I. Kuzminova, Maria E. Dmitrenko, Tatiana A. Hliavitskaya, Mathias Ulbricht

**Affiliations:** 1 Department of Analytical Chemistry, Institute of Chemistry, St. Petersburg State University, 7/9 Universitetskaya nab., 199034 St. Petersburg, Russia; s.ermakov@spbu.ru (S.S.E.); a.penkova@spbu.ru (A.V.P.); ai.kuzminova@mail.ru (A.I.K.); m.dmitrienko@spbu.ru (M.E.D.); 2 Institute of Physical Organic Chemistry, National Academy of Sciences of Belarus, 13 Surganov str., 220072 Minsk, Belarus; uf@ifoch.bas-net.by (A.V.B.); katyaburt@gmail.com (K.S.B.); hlyavitskaya1706@gmail.com (T.A.H.); 3 Lehrstuhl für Technische Chemie II, and Center for Water and Environmental Research (ZWU), University of Duisburg-Essen, 45141 Essen, Germany; mathias.ulbricht@uni-due.de

**Keywords:** membrane, polyelectrolyte, antifouling performance, ultrafiltration, water treatment

## Abstract

A novel method for one-step preparation of antifouling ultrafiltration membranes via a non-solvent induced phase separation (NIPS) technique is proposed. It involves using aqueous 0.05–0.3 wt.% solutions of cationic polyelectrolyte based on a copolymer of acrylamide and 2-acryloxyethyltrimethylammonium chloride (Praestol 859) as a coagulant in NIPS. A systematic study of the effect of the cationic polyelectrolyte addition to the coagulant on the structure, performance and antifouling stability of polysulfone membranes was carried out. The methods for membrane characterization involved scanning electron microscopy (SEM), atomic force microscopy (AFM), Fourier transform infrared spectroscopy (FTIR), contact angle and zeta-potential measurements and evaluation of the permeability, rejection and antifouling performance in human serum albumin solution and surface water ultrafiltration. It was revealed that in the presence of cationic polyelectrolyte in the coagulation bath, its concentration has a major influence on the rate of “solvent–non-solvent” exchange and thus also on the rate of phase separation which significantly affects membrane structure. The immobilization of cationic polyelectrolyte macromolecules into the selective layer was confirmed by FTIR spectroscopy. It was revealed that polyelectrolyte macromolecules predominately immobilize on the surface of the selective layer and not on the bottom layer. Membrane modification was found to improve the hydrophilicity of the selective layer, to increase surface roughness and to change zeta-potential which yields the substantial improvement of membrane antifouling stability toward natural organic matter and human serum albumin.

## 1. Introduction

Pressure-driven membrane separation processes, in particular ultrafiltration, are widely used for water treatment and purification, wastewater reclamation as well as the separation of liquid media in biotechnology, chemical, pharmaceutical, food and other industries. However, membrane fouling is still a serious problem limiting the potential of membrane technology [1]. Membrane fouling reduces the efficiency of the separation process and significantly increases the cost of separation. This is because of one or a combination for the following reasons; i.e., decrease of the flux through the membrane due to the formation of a gel-like diffusion barrier layer, biodegradation of the membrane-forming polymer and materials of the membrane module, the formation of concentrated populations of pathogenic microorganisms on the membrane surface, increasing energy consumption, the need for frequent chemical cleaning, reducing the usage time of the membrane and contamination of the filtrate [1,2,3,4]. The costs for cleaning consisting of costs for water, cleaning chemicals and wastewater are together with electricity by far the most dominating operation expenditures (OPEX) of membrane plants [4]. The main methods to prevent fouling before its occurrence are pre-treatment of the feed streams, chemical modification to improve the anti-fouling properties of a membrane, and optimization of the operational conditions [5].

Membrane surface properties such as chemical structure, hydrophilicity, roughness and charge are known to determine separation performance and fouling resistance [6,7,8]. An effective strategy for suppression of membrane fouling is to minimize attractive interactions between the membrane surface and components of the feed by chemical modification of the membrane surface which can be implemented by increasing membrane surface charge to promote electrostatic repulsion and hydrophilization to increase water–surface interaction [9,10]. The approaches utilized for the design of antifouling membranes involve (1) membrane polymer modification (pre-modification) [11], (2) blending of the membrane polymer with a modifying agent (additive) [12,13,14] and (3) surface modification after membrane preparation (post-modification) [15]. Grafting [5,7,15,16,17,18,19,20] and coating [21,22] are the most important and frequently utilized membrane surface modification methods.

Coating can be performed via the Langmuir–Blodgett technique, layer-by-layer deposition, copolymer adsorption, surfactant adsorption, salting-out effects and bio-adhesion [21]. Coatings feature more benefits compared to grafting due to the possibility to apply it to the existing installed membrane systems and modules via static or dynamic adsorption techniques. Moreover, the coating does not change the structural properties and mechanical strength of the membranes, less expensive additional equipment is needed and the modification time is usually not too long [22]. In contrast, grafting significantly changes membrane surface chemistry and physicochemical properties and requires excessive application of chemical solvents and various monomers [21,22]. However, both of these approaches are multi-step membrane formation techniques leading to the substantial increase of membrane cost.

Membrane modification by polyelectrolytes is a promising approach for the improvement of membrane antifouling performance as both charge and hydrophilicity of the selective layer can be increased at the same time which leads to a dramatic increase of antifouling stability [10]. The main disadvantage of this approach is that the performance of membranes modified by polyelectrolytes will strongly depend on the pH and ionic strength of the feed solution [10]. Modification of polymer membranes with polyelectrolytes can be used to produce membranes for reverse osmosis [5], nanofiltration [22,23,24], organic solvent nanofiltration [14], pervaporation [25] or ultrafiltration [26,27,28]. Polyelectrolytes are usually used in membrane modification through the surface coating method which involves static and dynamic layer-by-layer adsorption modification using pairs of oppositely charged polyelectrolytes [24,25,26,27] or formation of single polyelectrolyte layer in static or dynamic mode followed by cross-linking [28]. However, additional steps for membrane modification increase their cost, require additional equipment and are time- and labor-consuming.

In this work, a novel one-step technique for membrane modification via the addition of polyelectrolyte to the coagulation bath upon non-solvent induced phase separation (NIPS) is proposed. It was supposed that the addition of polyelectrolyte to the coagulation bath upon membrane formation via NIPS will result in the immobilization of polyelectrolyte macromolecules to the membrane selective layer due to their in-diffusion to the nascent polymer film. Polyelectrolyte macromolecules are expected to be fixed in the membrane matrix when polymer precipitates leading to the increase of charge and hydrophilicity of the membrane selective layer which will yield the improvement of antifouling performance. The composition of the coagulation bath is known to have a powerful impact on membrane structures by adjusting the liquid–liquid demixing in NIPS [29].

According to a literature review, only a few works deal with the addition of polymer [30] and polyelectrolytes [31,32] to the coagulation bath in NIPS. The method for preparation of polysulfone hollow fiber membrane with high antifouling performance is proposed which involves the use of aqueous solutions of polyvinylpyrrolidone (PVP K-30; 1–5 wt.%) as a bore fluid in the dry-jet wet-spinning process. It is reported that the proposed method of modification allows a significant improvement of the antifouling performance of the membrane without changing the technology of the hollow fiber membrane spinning [30]. Carboxylated cardo poly(arylene ether ketone) (PAEK-COOH) was activated by adding 1-(3-dimethylaminopropyl)-3 ethylcarbodiimide hydrochloride and N-hydroxysuccinimide to the casting solution in the mixed solvent (N-methyl-2-pyrrolidone and 1,4-dioxane) to prepare ultrafiltration membranes [31]. Then, the activated polymer film was immersed into a polyethyleneimine (PEI) aqueous solution that served as a coagulation bath for taking the one-step chemical reaction-involved phase inversion process. During the in situ cross-linking of the activated carboxylic acid group and PEI along with the phase inversion process, the PEI molecules had been covalently bound onto membrane surface and resulted in the increase of membrane hydrophilicity. The prepared membrane was reported to have high antifouling performance in dye removal from textile wastewater [31]. A reaction-enhanced surface segregation method was explored for in situ construction of the antifouling separation layer on the membrane surface [32]. PVP was used as the surface segregation agent in the casting solution and polyacrylic acid (PAA) was used as the additive in the coagulation bath. During the membrane formation process, PVP and PAA spontaneously migrate to the surface of the polyvinylidene fluoride (PVDF) support in the opposite direction and form a crosslinked separation layer based on the hydrogen bonding interaction. The resultant layer has smaller effective pore size and higher hydrophilicity compared with the PVDF control membrane, which renders enhanced antifouling and separation performance in the ultrafiltration of oil–water emulsion and bovine serum albumin solution [32].

It is worth noting that, according to the literature review, the effect of the addition of polyelectrolyte to the coagulation bath upon NIPS on membrane structure and performance has not been studied yet. In this work, a commercial flocculant, a cationic polyelectrolyte, copolymer of acrylamide and 2-acryloxyethyltrimethylammonium chloride (Praestol 859, *M*_n_ = (10–14) × 10^6^ g∙mol^−1^, content of charged groups is 90%) was utilized for the first time as an additive to the coagulation bath in membrane formation by NIPS. Commercial flocculants based on polyacrylamide are widely used for purification and disinfection of drinking water. Moreover, they have found applications in chemical, petrochemical and pulp industries [33]. They are relatively cheap, available polyelectrolytes produced at a large scale [34,35,36,37].

The goal of this study was to reveal the effect of the cationic polyelectrolyte addition to the coagulant on the structure, performance and antifouling stability of polysulfone ultrafiltration membranes.

## 2. Materials and Methods

### 2.1. Materials

**Figure 1:**
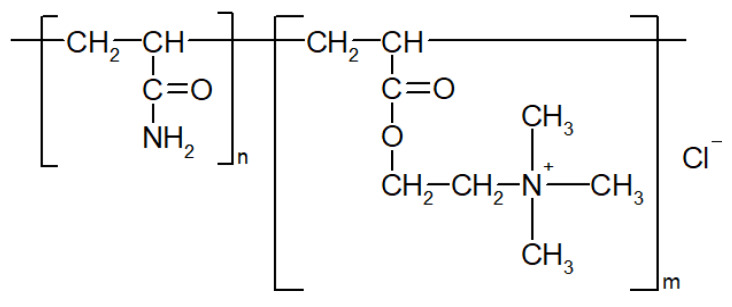
Chemical formula of cationic polyelectrolyte Praestol 859.

Polysulfone (PSF, Ultrason S 6010, *M*_n_ = 55,000 g∙mol^−1^, BASF, Ludwigshafen, Germany) was utilized as a membrane-forming polymer for the preparation of flat sheet membranes. Polyethylene glycol (*M*_n_ = 400 g∙mol^−1^, BASF, Ludwigshafen, Germany) was used as an additive to the casting solution, N,N-dimethylacetamide (DMAc, BASF, Ludwigshafen, Germany) was used as a solvent. Distilled water and aqueous solutions of commercial flocculant which is utilized for water treatment (Praestol 859, Ashland Inc., Covington, KY, USA) based on polyacrylamide were used as a coagulation bath upon membrane preparation via non-solvent induced phase separation (NIPS). Praestol 859 is a cationic polyelectrolyte, copolymer of acrylamide and 2-acryloxyethyltrimethylammonium chloride (*M*_n_ = (10–14) × 10^6^ g∙mol^−1^, content of charged groups is 90%). The chemical formula is presented in Figure 1.

Human serum albumin (HSA, *M*_w_ = 66,400 g∙mol^−1^, *pI* = 4.6, Sigma-Aldrich, St. Louis, MO, USA) was used as a test substance to estimate membrane separation performance. To test the antifouling performance of the developed membranes with HSA solutions as a feed phosphate buffer (K_2_HPO_4_–KH_2_PO_4_) was used to adjust pH to 7.0, glycine–HCl buffer, acetate buffer and glycine–NaOH buffer were used to adjust the pH to 3.2, 4.6 and 9.6, respectively.

### 2.2. Preparation of PSF Casting Solution

To prepare the casting solution, 18 wt.% PSF, 15 wt.% PEG-400 and 67 wt.% DMAc were added to a round-bottom flask and mixed for 5 h at 120 °C with an overhead stirrer at 600 rpm. All the components were used without further purification. Thereafter, the prepared casting solution was cooled down at room temperature and left overnight to remove air bubbles.

### 2.3. Preparation of Praestol 859 Aqueous Solutions

To prepare Praestol 859 aqueous solutions with concentrations 0.05 wt.%, 0.1 wt.%, 0.2 wt.% and 0.3 wt.% the predetermined amount of cationic polyelectrolyte was added to 2 L of distilled water and mixed with overhead stirrer for 8 h at room temperature at 100–200 rpm. Prepared aqueous solutions of Praestol 859 were used as a coagulation bath for membrane formation via NIPS the next day after preparation. Praestol 859 aqueous solutions were filtered using a Bunsen flask, Buchner funnel, filter paper and a water jet pump prior to application.

### 2.4. Viscosity Studies

Viscosity of the Praestol 859 aqueous solutions at different temperatures (25 °C, 40 °C, 50 °C, 60 °C) was measured by Ostwald viscometer (capillary diameter *d* = 0.73 mm was used for 0.05 wt.% aqueous Praestol 859 solution; *d* = 1.77 mm for 0.1–0.3 wt.% aqueous Praestol 859 solutions; *d* = 2.37 mm for 0.5 wt.% aqueous Praestol 859 solution).

### 2.5. Coagulation Value of Praestol 859 Aqueous Solutions

To study the effect of Praestol 859 addition to the coagulation bath on the thermodynamics of the phase separation of PSF casting in DMAc coagulation values (CVs) of 0.05–0.3 wt.%, Praestol 859 aqueous solutions were determined by titration method. CV is the amount (g) of the coagulant needed to cause the phase separation of 100 mL (1 dL) of 1 wt.% polymer solution. CV is the indication of polymer solution tolerance toward coagulants. The CVs were determined by titration of 100 mL of 1 wt.% PSF solution in DMAc by distilled water and 0.05–0.3 wt.% Praestol 859 aqueous solutions with constant stirring of the titrated solution on a magnetic stirrer at room temperature. Three different samples of polymer solution were titrated and the average CV was calculated. The cloud point was recorded if, after 40 min of stirring, the solution remained cloudy.

### 2.6. Preparation of Membranes

To obtain PSF flat-sheet membranes by NIPS, a casting solution at 25 °C was applied to a rough (average profile roughness, *R*_a_ = 1.4–1.6 μm) or smooth glass plate using a casting blade with the thickness of 250 μm. The glass plate with the applied layer of a casting solution was immediately immersed in the coagulation bath. Distilled water and aqueous solutions of Praestol 859 of different concentrations (0.05 wt.%, 0.1 wt.%., 0.2 wt.% and 0.3 wt.%) at different temperatures (25 °C, 40 °C, 60 °C and 70 °C) were used as coagulants for membrane preparation via NIPS. Conditions for membrane preparation and membrane abbreviations are presented in Table 1. The casting solution composition was 18 wt.% PSF, 15 wt.% PEG-400 and 67 wt.% DMA for all membranes. Rough and smooth glass plates were applied for membrane preparation to study the effect of glass plate roughness on the membrane structure and performance. The prepared membranes were kept in distilled water at least 24 h before usage.

### 2.7. Determination of the Time of Membrane Formation

The time of membrane formation in NIPS was determined as the time from the moment of immersion of a nascent polymer film into the coagulation bath to the moment when the precipitated membrane completely separates from the plate. Membrane formation time was determined for different coagulation bath compositions at *T* = 25 °C, i.e., distilled water, as well as 0.05 wt.%, 0.1 wt.%, 0.2 wt.% and 0.3 wt.% Praestol 859 aqueous solutions. Rough and smooth glass plates were used for membrane preparation at all coagulation bath compositions to evaluate the influence of the glass plate roughness on the membrane formation time.

### 2.8. Membrane Separation Performance Studies

A procedure for membrane performance studies is reported in the previous works [14,38]. Briefly, pure water flux (PWF, L·m^−2^·h^−1^) and flux of model solutions (0.5 wt.% HSA solution in different buffers) were tested using stirred ultrafiltration cell with effective membrane area 22.4 cm^2^ equipped with a magnetic stirrer. Measurements were carried out at transmembrane pressure 1 bar, T = 25 °C and rotation speed 200 rpm. The membrane sample was pressurized for 30 min upon distilled water filtration at 1 bar prior to each measurement. Thereafter, PWF was recorded and distilled water was then replaced in the cell by 0.5 wt.% HSA solution in different buffers. After 30 min of filtration, the flux of model solutions was determined. To determine membrane rejection, a UV–Vis spectrophotometer (Metertech UV–VIS SP 8001, Taipei, Taiwan) at a wavelength of 280 nm was used to measure HSA contents in the feed and permeate.

### 2.9. Antifouling Performance Studies

To study membrane antifouling performance, 0.5 wt.% HSA solutions in different buffer solutions were used (Table 2).

The procedure applied to study membrane antifouling stability is reported elsewhere [14,39]. Briefly, the membrane sample was placed in the stirred ultrafiltration cell and distilled water was filtered at 1 bar for 30 min and pure water flux was determined. Thereafter, 0.5 wt.% HSA solution was filtered for 1 h and flux was measured every 15 min. After HSA ultrafiltration, pure water flux was determined again. Thereafter, the fouled membranes were washed by distilled water for 0.5 h in the ultrafiltration mode and the PWF of the washed membranes (JWF) was measured. This procedure was repeated 2 times. Flux recovery ratio (FRR), reversible flux decline ratio (DR_r_), irreversible flux decline ratio (DR_ir_) and total flux decline ratio (DT) were calculated according to [39].

### 2.10. FTIR Studies

The composition of the membrane selective layer was investigated by a frustrated total internal reflection method using Fourier transform infrared spectroscopy (FTIR)-spectrometer Nicolet Is50 (ThermoFisher Scientific, Waltham, MA, USA) in the frequency range 400–4000 cm^−1^, accuracy 0.01 cm^−1^. Sample preparation included drying of the membranes at ambient conditions for 3 days.

### 2.11. Membrane Structure Studies

The structure of the flat sheet membranes was studied via scanning electron microscopy (SEM) and atomic force microscopy (AFM). Membrane samples were impregnated by 20% glycerol solution for 1 h and dried at room temperature for 3 days prior to SEM and AFM studies. Impregnation with glycerol solution was carried out in order to preserve membrane pore structure upon drying.

The membrane cross-section structure was studied using a scanning electron microscope (SEM) LEO 1420 (LEO Electron Microscopy Inc., Thornwood, NY, USA). The membrane cross-sections were prepared by fracturing the membrane samples in liquid nitrogen followed by gold layer deposition via cathode sputtering in a vacuum setup (EMITECH 550X, Quorum Technologies Ltd., Laughton, UK).

The structure of the surface of the samples was studied on an HT- 206 atomic force microscope with standard silicon cantilevers with a rigidity of 3 N∙m^−1^ (MikroMash, Wetzlar, Germany).

### 2.12. Contact Angle Measurements

Water contact angle (θ, °) was determined by the attached air bubble technique using the LK-1 goniometer (“Otkrytaya nauka”, Krasnogorsk, Russia). The advantage of this method is the complete hydration of the surface of the membrane under investigation. Thus, it can be expected that the interfacial energy between water and the membrane will not change during the measurement of the geometric parameters of the air bubble at the membrane–water interface [14,39]. Measurement of contact angles was made in a three-phase system consisting of water, the membrane surface and an air bubble.

### 2.13. Determination of Zeta-Potential of the Membrane Selective Layer

Zeta-potential of membrane selective layer was determined using electrokinetic analyzer SurPASS 2 (Anton Paar, Graz, Austria). The membrane sample was kept in MilliQ water for 24 h before tests. The sample holder for discs was used; the gap height between two discs of the membrane sample was set 100–106 μm. Aqueous potassium chloride solution in MilliQ water with a concentration of 1 mmol·L^−1^ was used as an electrolyte. Zeta-potential was measured in the pH range from 3.0 to 10.0 with steps of 0.5–0.7 pH units.

### 2.14. Surface Water Analysis and Ultrafiltration Study

Surface water (Slepianski Channel, Minsk, Belarus) was used as a feed for ultrafiltration experiments. Iron content in the feed surface water and permeate was determined by an inductively coupled plasma atomic emission spectrometer (Vista PRO, Varian, Palo Alto, CA, USA). Total organic carbon was determined using TOC-LCSH/CPH (Shimadzu, Kyoto, Japan). Feed surface water and permeate turbidity was determined using 2100 AN turbidimeter manufactured by HACH (Loveland, CO, USA) with a tungsten incandescent lamp as a light source and a filter with a wavelength of 860 nm. The optical density of feed surface water and permeate was measured using Metertech UV/VIS SP 8001 spectrophotometer (Metertech, Taipei, Taiwan) at a wavelength of 400 nm.

The procedure was the following: The membrane sample was mounted in the stirred ultrafiltration cell with a volume of 400 mL and membrane effective area 24.6 cm^2^. The membrane was compacted at 1 bar for 30 min upon ultrafiltration of distilled water. Thereafter, surface water was placed in the cell and filtered at a transmembrane pressure of 1 bar, 20 °C and stirrer speed 200 rpm. The volume of permeate was recorded every 30 s. The ultrafiltration was carried out for 1 h and the normalized flux (*J*_norm_) was calculated according to Equation (1):

(1) $$J_{\mathrm{norm}}=\frac{J}{J_{0}},$$

where *J*_0_ is the flux in the initial moment of filtration, L·m^−2^·h^−1^, and *J* is the flux determined at a certain time interval of filtration, L·m^−2^·h^−1^.

## 3. Results

**Figure 2:**
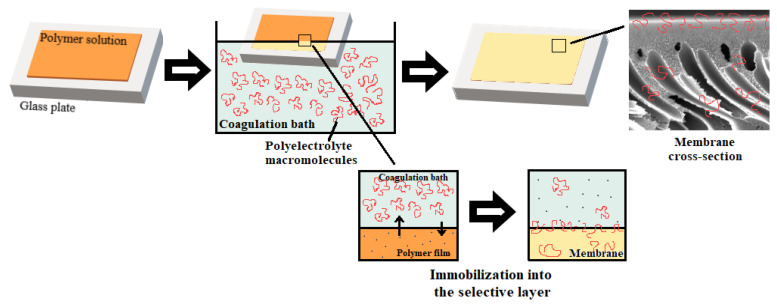
Preparation of membranes by non-solvent induced phase separation (NIPS) using the aqueous solution of polyelectrolyte as a coagulant.

The idea of the study was that the addition of polyelectrolyte to the coagulation bath upon membrane formation via NIPS will yield the immobilization of the polyelectrolyte macromolecules into the membrane selective layer (Figure 2). It was supposed that controlled variation in the composition of the coagulation bath using polyelectrolyte allows controlled changes in the precipitation conditions for polymer membranes and thus also in the chemical composition of the membrane selective layer. It was expected that the immobilization of polyelectrolyte macromolecules at the stage of membrane formation will fix polyelectrolyte macromolecules in the membrane matrix upon polymer precipitation and solidification (Figure 2). Immobilization of polyelectrolyte macromolecules was expected to increase hydrophilicity and impart a charge to the membrane selective layer which will improve membrane antifouling performance. Praestol 859 was selected for membrane modification because of the high content of positively charged groups (90%, Figure 1), as well as relative cheapness, availability and large-scale production [34,35,36,37].

There are two possible factors affecting the structure and performance of polymer membranes when polyelectrolyte additives are introduced to the coagulation bath: (1) increase of casting solution tolerance toward coagulant, and (2) increase of coagulant viscosity. When casting solution tolerance toward coagulant increases, polymer precipitation occurs in milder conditions compared to the case when only water is used as a coagulant. It means that the addition of polyelectrolyte will cause more non-solvent to be required for inducing demixing. To check the first assumption, the coagulation values (CVs) of distilled water and 0.05–0.3 wt.% Praestol 859 aqueous solution for 1 wt.% of PSF solution in DMAc were determined by titration method. It was found that the CV of distilled water for 1 wt.% PSF solution in DMAc is 4.0 g·dL^−1^ and addition of 0.05–0.3 wt.% Praestol 859 does not change CV. Thus, it was revealed that the addition of cationic polyelectrolyte to the coagulation bath does not influence the thermodynamics of casting solution-phase separation and the PSF casting solution tolerance toward coagulant does not change. So, the main factor affecting the structure and performance of polymer membranes when Praestol 859 is added to the coagulation bath is an increase of coagulant viscosity. The increase in the coagulant viscosity results in the decrease of the “solvent–non-solvent” exchange rate in the NIPS process; it means that the in-diffusion rate of the coagulant into the nascent polymer film and out-diffusion rate of the solvent out of the nascent polymer film decrease.

### 3.1. Time of Membrane Formation

The dependence of membrane formation time on the cationic polyelectrolyte concentration in the coagulation bath was studied (Table 3). It was found that the application of rough glass plate for membrane formation leads to longer times for membrane formation under all conditions. This is due to the better adhesion of polymer film between surface irregularities (elements of surface roughness) of the glass plate. Moreover, the release of the nascent membrane from support and thus coagulation from the back side is prevented with a rough plate. Furthermore, an increase of membrane formation time with the increase of Praestol 859 concentration in the coagulation was observed (Table 3). A decrease in the exchange rate is expected which yields the formation of a thicker selective layer, eventually resulting in a decrease of membrane permeability. The influence of Praestol 859 concentration on coagulation bath viscosity was studied in more detail (Section 3.2).

### 3.2. Viscosity Studies

**Figure 3:**
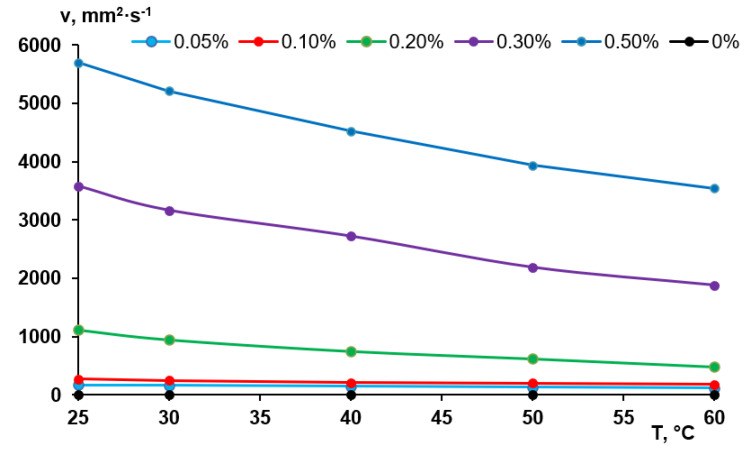
Viscosity of the aqueous solutions of Praestol 859 at different concentrations vs. temperature.

According to the abovementioned considerations, the viscosity of the coagulation bath is an important factor for membrane formation via NIPS especially when polymers with very high molecular weight are added to the coagulant. The viscosity of the Praestol 859 solutions in distilled water determined using a capillary Ostwald viscometer is presented in Figure 3. It was revealed that aqueous solutions of Praestol 859 feature very high viscosity at low polyelectrolyte concentrations due to very high molecular weight (Figure 3). When polyelectrolyte concentration increases, the viscosity dramatically increases. For instance, when polyelectrolyte concentration increases from 0.05 wt.% to 0.5 wt.% (10 times) the viscosity of the Praestol 859 aqueous solution at *T* = 25 °C increases from 170 to 5703 mm^2^·s^−1^ (33.5 times).

**Figure 4:**
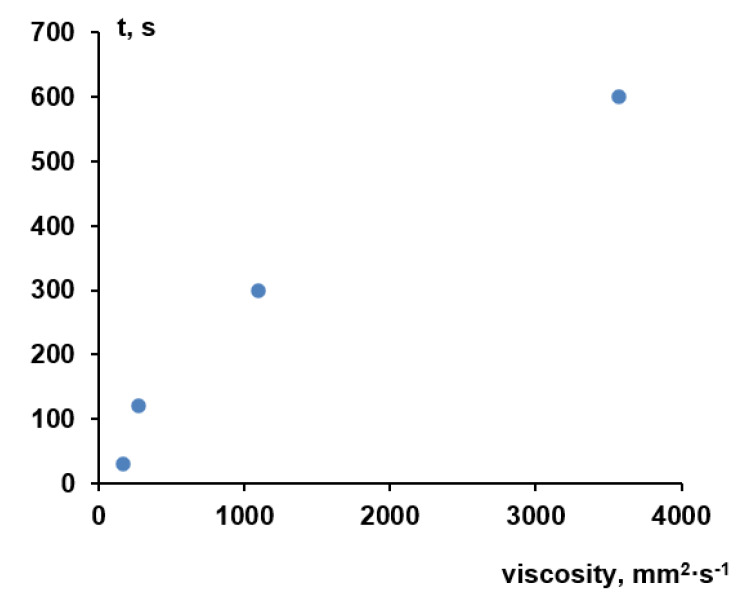
The dependence of membrane formation time on the viscosity of aqueous Praestol 859 solutions used as a coagulation bath (rough plate, *T* = 25 °C).

It is worth noting that the viscosity of the aqueous solutions of Praestol 859 is very sensitive to the change of temperature. For instance, when temperature increases from 25 °C to 60 °C the viscosity of 0.5 wt.% aqueous solution of Praestol 859 decreases from 5703 mm^2^·s^−1^ to 3543 mm^2^·s^−1^ (1.61 times). The data obtained prove that the viscosity of cationic polyelectrolyte aqueous solutions dramatically depends on even very small concentration change and temperature. It means that the change of polyelectrolyte concentration in the coagulation bath and coagulation bath temperature will lead to the change of the viscosity of coagulant which will largely affect the kinetics of membrane formation. Indeed, a systematic correlation between membrane formation time and viscosity is observed (Figure 4).

It should be taken into account that membrane formation time as determined in this study is a global measure of kinetic processes during NIPS and will not reflect all mechanistic details; what happens at the interface upon contact between cast film and coagulant is certainly not fully covered. In particular, the big difference in viscosity between water and 0.05 wt.% Praestol 859 (about 30 times higher) does not fit well into the correlation (time for membrane formation is only two times longer; value for water is not included in Figure 4). Nevertheless, the data clearly reveal that in presence of Praestol 859, its concentration has a major influence on the rate of “solvent–non-solvent” exchange and thus also on the rate of phase separation.

The relationship between the overall rate of membrane formation (compare Table 3) and membrane structure will be further analyzed and discussed in Section 3.4.

### 3.3. Composition of the Membranes

**Figure 5:**
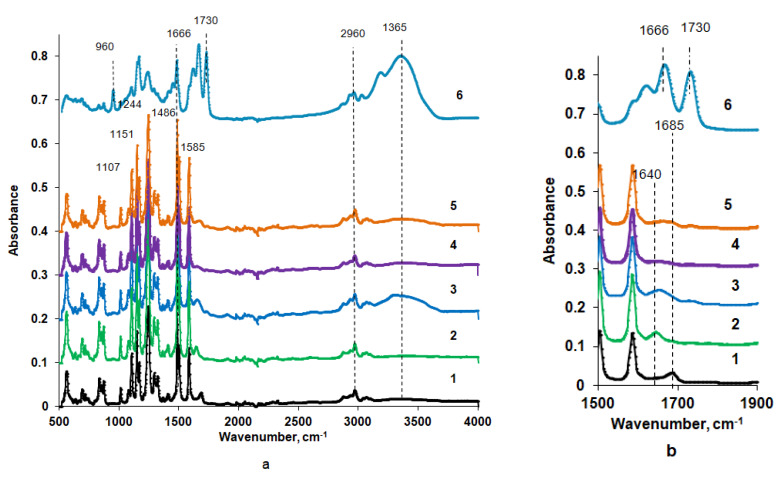
Fourier transform infrared spectroscopy (FTIR) spectrum (**a**) and fragment of the spectrum (**b**) of the polysulfone (PSF) membrane selective (1–3 and 5) and bottom (4) layer; Praestol 859 in a coagulation bath, wt.%: 1—0, *T* = 60 °C (PSF60); 2—0.2, *T* = 60 °C (B-0.2-60); 3—0.3, *T* = 60 °C (B-0.3-60); 4—0.3, *T* = 60 °C (B-0.3-60), bottom layer; 5—0.3; *T* = 25 °C (B-0.3-25); 6—Praestol 859 powder.

FTIR spectra of the surface of the selective and bottom layers of the reference PSF membrane prepared with water as a coagulation bath and membranes, prepared using Praestol 859 aqueous solutions as coagulants, are presented in Figure 5.

It was revealed that vibrations of the PSF membrane matrix dominate in the spectra of both reference and modified membranes, which makes it difficult to detect vibrations of Praestol 859 macromolecules immobilized in the selective layer due to their very low concentration in the membrane matrix. Stretching vibrations of the SO_2_ group are presented by the absorption band at 1294 cm^−1^ (1–5 in Figure 5). The absorbance peak at 1151 cm^−1^ is assigned to the –C–SO_2_–C– symmetric stretching [40]. Absorbance peaks at 1585, 1504, 1486 and 1107 cm^−1^ are attributed to the vibrations of the PSF aromatic ring (C=C stretching) [40]. The peak at 1244 cm^−1^ is attributed to the aromatic ether bond (–C–O–C–) of PSF [34]. Stretching vibrations of the C–H bond of the methyl groups of PSF are observed at 2860 and 2960 cm^−1^ (1–5 in Figure 5). The peak at 1685 cm^−1^ for the PSF60 membrane is attributed to the contributions from C=O and N–C stretching vibrations from traces of the solvent DMAc left in the membrane matrix despite the washing in distilled water (1 in Figure 5).

The spectrum of pure Praestol 859 powder is also presented in Figure 5 (Curve 6). A broad absorbance band in the range of 3500–3150 cm^−1^ with the maximum at 3365 cm^−1^ corresponds to the NH_2_ group involved in the hydrogen bond formation of Praestol 859 acrylamide units. The peak at 1730 cm^−1^ is assigned to the C=O stretching vibrations of ester bonds from 2-acryloxyethyltrimethylammonium units of copolymer. The peak at 1666 cm^−1^ is attributed to the amide I (C=O stretching vibrations). The absorbance band at 960 cm^−1^ is due to the quaternary ammonium. The peaks at 2960 and 2930 cm^−1^ are attributed to symmetric and asymmetric –C–H bond stretching vibrations. The absorbance peak at 1486 cm^−1^ is assigned to the δ(–CH_2_–N^+^(CH_3_)_3_) [41].

A change of the shape of the broad peak at 1685 cm^−1^ observed in the spectrum of the reference PSF60 membrane and a shift of its maximum from 1685 cm^−1^ to 1640 cm^−1^ was observed for the FTIR spectra of the selective layer surfaces of membranes prepared using aqueous solutions of Praestol 859 as a coagulant (2, 3 and 5 in Figure 5). The broad absorbance band in the range of 1610–1670 cm^−1^ with the maximum at 1640 cm^−1^ is attributed to the overlapping of amide I (mainly C=O stretching vibrations) in the Praestol 859 acrylamide group and amide group from DMAc traces left in membrane matrix (2, 3 and 5 in Figure 5). This proves the immobilization of Praestol 859 macromolecules in the membrane selective layer. It was found that with the increase of Praestol 859 concentration in the coagulation bath up to 0.3 wt.%, the broad band in the range of 3500–3150 cm^−1^ with the maximum at 3365 cm^−1^ appears in the spectra of B-0.3-60 and B-0.3-25 membranes (3 and 5 in Figure 5). As was mentioned above, this peak is assigned to the stretching vibrations of NH_2_-groups of Praestol 859 acrylamide unit involved in hydrogen bonding.

Comparison of the spectra of the surface of the bottom and selective layers of B-0.3-60 membrane allows concluding that cationic polyelectrolyte macromolecules are mainly immobilized on the surface of the selective layer (compare 3 and 4 in Figure 5), which during NIPS is first in contact with the coagulation bath (compare Figure 1). This is due to their high molecular weight, which hinders their diffusion inside the nascent polymer film upon membrane formation. According to the analysis of the spectra of the selective layer surface of membranes prepared using 0.3 wt.% aqueous Praestol 859 solution as a coagulant at 25 °C and 60 °C (B-0.3-25 and B-0.3-60), it can be concluded that increase of temperature results in the increase of the amount of immobilized Praestol 859 macromolecules, which is due to the coagulant viscosity decrease (compare Section 3.2). Decrease of coagulant viscosity makes diffusion of the polyelectrolyte into the as cast polymer film and, consequently, its immobilization upon membrane formation via NIPS more effective.

### 3.4. Membrane Structure Studies

**Figure 6:**
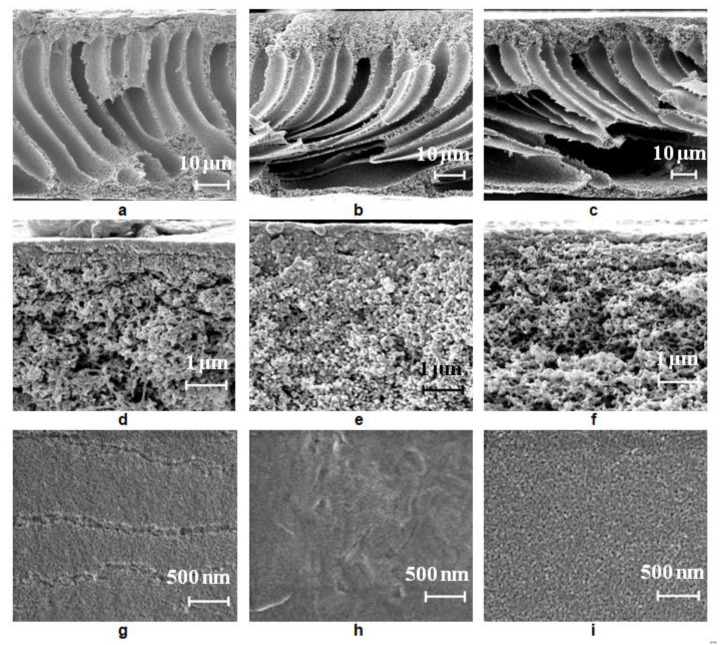
Scanning electron microscopy (SEM) images of the PSF membrane cross-section (**a–c**), selective layer (**d**–**f**), surface of the selective layer (**g**–**i**), prepared using a smooth glass plate, coagulation bath, *T* = 25 °C: (**a**,**d**,**g**) distilled water; (**b**,*e*,**h**) 0.05 wt.% Praestol 859 aqueous solution; (**c**,**f**,**i**) 0.3 wt.% Praestol 859 aqueous solution.

**Figure 7:**
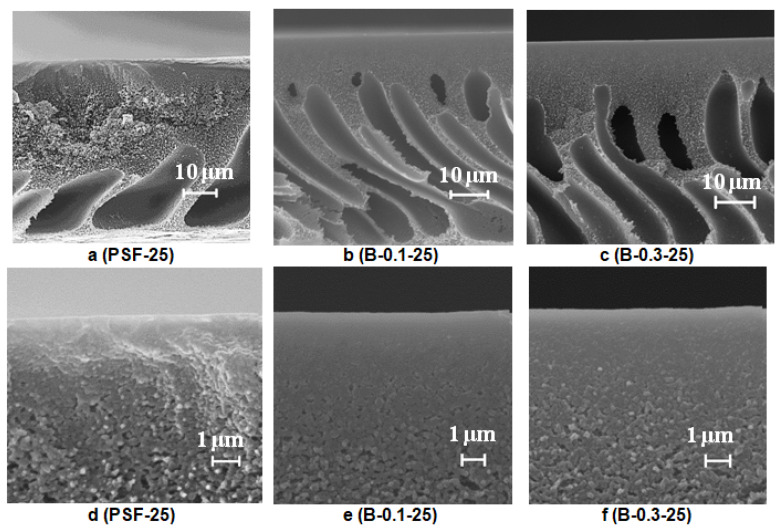
SEM images of the PSF membrane cross-section (**a**–**c**) and selective layer (**d**–**f**) prepared using a rough glass plate, Praestol 859 concentration in the coagulation bath (*T* = 25 °C), wt.%: (**a**,**d**) 0; (**b**,**e**) 0.1; (**c**,**f**) 0.3.

**Figure 8:**
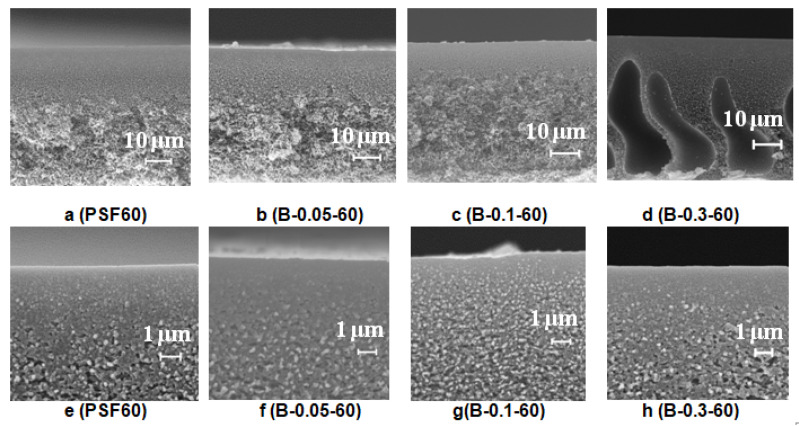
SEM images of the PSF membrane cross-section (**a**–**d**) and selective layer (**e**–**h**) prepared using a rough glass plate, Praestol 859 concentration in the coagulation bath (*T* = 60 °C), wt.%: (**a**,**e**) 0; (**b**,**f**) 0.05; (**d**,**h**) 0.3.

**Figure 9:**
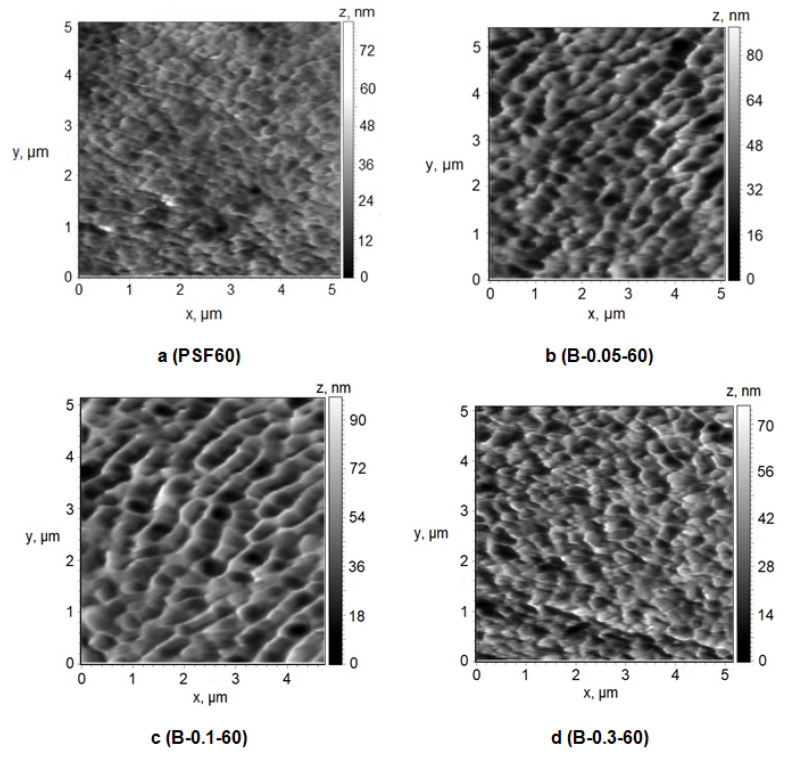
Atomic force microscopy (AFM) images of the surface of the selective layer, Praestol 859 concentration in the coagulation bath (*T* = 60 °C), wt.%: (**a**) 0; (**b**) 0.05; (**c**) 0.1; (**d**) 0.3.

**Figure 10:**
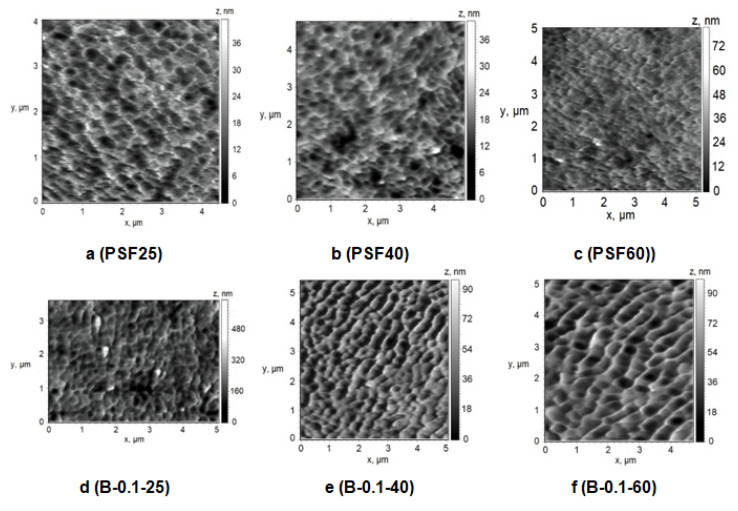
AFM images of the surface of the selective layer of membranes prepared at coagulation bath temperature *T* = 25 °C (**a**,**d**), *T* = 40 °C (**b**,**e**), *T* = 60 °C (**c**,**f**) and Praestol 859 concentration in the coagulation bath: (**a**–**c**) 0 wt.%.; (**d**–**f**) 0.1 wt.%.

The effect of the addition of the cationic polyelectrolyte Praestol 859 to the coagulation bath on membrane structure was studied using scanning electron microscopy and atomic force microscopy (Figure 6, Figure 7, Figure 8, Figure 9 and Figure 10). Moreover, the influence of the coagulation bath temperature (25 °C and 60 °C) and type of support (smooth and rough glass plate) upon membrane formation via NIPS was studied. It is known that type of support (especially roughness) upon membrane formation can significantly influence membrane structure and performance [42].

#### 3.4.1. SEM Studies

##### Effect of Cationic Polyelectrolyte Concentration in the Coagulation Bath on Membrane Structure

It was found that an increase of Praestol 859 concentration in the coagulation bath strongly affects the coagulation time of cast polymer film (Table 3); this is due to the significant increase of coagulant viscosity (Section 3.2).

First, the influence of cationic polyelectrolyte concentration in the coagulation bath on the structure of membranes prepared using a smooth glass plate was studied.

The structure of the reference PSF membrane prepared using distilled water as a coagulant is a typical anisotropic morphology with a thin selective layer and finger-like macrovoids formed upon the instantaneous demixing mechanism (Figure 6a,d). When even a low concentration of Praestol 859 (0.05 wt.%) is added to the coagulation bath, the viscosity increase is very large (from 0.89 mm^2^·s^−1^ for water to 170.2 mm^2^·s^−1^ for 0.05 wt.% Praestol 859 solution; Figure 3) which results in the increase of membrane formation time (Table 3). It was found that with the addition of 0.05 wt.% Praestol to the coagulation bath (*T* = 25 °C), upon membrane preparation using a smooth glass plate, large elongated macrovoids move further from the membrane selective layer (Figure 6a,b). It was revealed that the structure of the transitional layer (located in the vicinity of the selective layer) changes from the sponge-like for initial the PSF25 membrane to a globular one for the B-0.05-25 membrane prepared using a smooth glass plate (Figure 6d,e). It was shown that the selective layer becomes denser; pore size decreases and cannot be detected by SEM (Figure 6d,e,g,h). When 0.3 wt.% Praestol 859 is added to the coagulation bath, the viscosity of the coagulant dramatically increases (Figure 3). It yields the increase of membrane formation time up to 2 min (Table 3). It is supposed that membrane formation occurs via the delayed demixing mechanism. When the cationic polyelectrolyte concentration increases up to 0.3 wt.% in the coagulation bath, macrovoids were found to move closer to membrane selective layer, and the size of the large irregular macrovoids near the membrane bottom layer increased (Figure 6c,f,i). The thickness of the selective layer decreases and a spongy transitional layer is formed similar to the initial PSF25 membrane (Figure 6d,f). The pore size of the selective layer was found to increase compared to the pore size of the selective layer of B-0.05-25 membrane due to the increase of casting solution tolerance toward the coagulant and decrease of the rate of membrane-forming polymer precipitation (Figure 6h,i). It is worth noting that the pore size of the selective layer for B-0.3-25 membrane prepared using a smooth glass substrate was revealed to be larger compared to the PSF25 membrane (Figure 6h,i). The formation of large irregular macrovoids near the bottom layer in the case of both B-0.05-25 and B-0.3-25 is considered to be due to the uneven diffusion of the coagulant to the cast polymer film (Figure 6b,c). Praestol 859 is a very high-molecular-weight polyelectrolyte. At the beginning of the NIPS process via delayed demixing mechanism membrane-forming polymer coagulation occurs and the gel-like partly precipitated selective layer is formed. Praestol 859 aqueous solution which is used as a coagulant has to diffuse through this partly precipitated gel region inside the polymer cast film. This diffusion is expected to be not uniform because of the high molecular weight of cationic polyelectrolyte and formation of macromolecule associates in the solution which results in the fluctuations of concentration. Moreover, partly precipitated gel-like polymer-network formed in NIPS via delayed demixing mechanism may hinder the diffusion of large cationic polyelectrolyte macromolecules. It is worth mentioning that formation of large macrovoids in the membrane matrix is an undesirable phenomenon because it decreases membrane mechanical strength.

##### Effect of Glass Support Type on Membrane Structure

It was found that when a rough glass plate is used for membrane preparation, the membrane matrix becomes more uniform compared to the membrane prepared using a smooth plate (Figure 6a and Figure 7a). It was shown that as the number of macrovoids decreases, macrovoids become thicker and move further from the selective layer compared to the membrane prepared using a smooth glass plate (Figure 6a and Figure 7a). Due to polymer solution adhesion, the rough surface of the glass substrate sets a pattern for the formation of membrane structure in the vicinity of the membrane bottom layer which prevents large irregular macrovoid formation. It was revealed that macrovoids in the membrane matrix change their shape from more symmetrical elliptic to elongated one upon 0.1 wt.% Praestol 859 addition to the coagulation bath (Figure 7a,b). Moreover, the macrovoids were observed to move closer to the selective layer upon Praestol 859 addition to the coagulant compared to the initial PSF25 membrane (Figure 7a). When the concentration of Praestol 859 in the coagulation bath increased up to 0.3 wt.% (*T* = 25 °C), elongated macrovoids were found to become thicker (Figure 7c).

It was revealed that when Praestol 859 concentration in the coagulation bath increased up to 0.1–0.3 wt.% and the structure of the membrane matrix became denser and less porous compared to the structure of initial PSF25 membrane prepared using a rough glass plate (Figure 7a–c).

It was found that the addition of cationic polyelectrolyte to the coagulant yields the formation of a denser and thicker selective layer with a more uniform structure compared to the initial PSF membrane (Figure 7d–f). However, the thickness of the selective layer of the membranes prepared using 0.1 wt.% cationic polyelectrolyte solution was shown to be larger compared to the membrane prepared using 0.3 wt.% solution (Figure 7e,f).

The membrane structure prepared using a rough glass plate was found to be more regular and uniform with a smaller number of macrovoids which is preferably for operating under pressure. So, this type of structure was selected for further studies.

##### Effect of Coagulation Bath Temperature

To study the effect of coagulation bath temperature on membrane structure reference, PSF membrane and membranes modified with cationic polyelectrolyte solution of different concentrations were prepared using rough glass plate at a coagulation bath temperature of 60 °C.

It was revealed that an increase of the coagulation bath temperature leads to the formation of a spongy macrovoid-free membrane structure both for the reference PSF60 membrane prepared using distilled water as a coagulant and, for the membranes prepared using 0.05–0.1 wt.% Praestol 859 aqueous solutions as coagulants (Figure 8). However, upon the increase of cationic polyelectrolyte concentration in the coagulant up to 0.3 wt.%, large macrovoids appear in the membrane matrix (Figure 8d). When cast polymer film is placed in the hot coagulation bath, the temperature of the polymer solution increases. Temperature increase results in the increase of casting solution tolerance to the coagulant especially for cationic polyelectrolyte aqueous solution which causes delayed demixing, usually attributed to the macrovoid-free structure formation. On the other hand, temperature increase yields a significant decrease of Praestol 859 solution viscosity which increases the “solvent–non-solvent” exchange rate upon membrane formation via NIPS (Figure 3 and Figure 4). The balance of these two factors affects the membrane structure. Upon the increase of the Praestol 859 concentration in the coagulation bath up to 0.3 wt.%, the coagulant bath viscosity (*T* = 60 °C) dramatically increases 10.5 times compared to 0.1 wt.% Praestol 859 (from 179 mm^2^·s^−1^ up to 1879 mm^2^·s^−1^). However, large macrovoids appear in the membrane matrix due to the high heterogeneity of the 0.3 wt.% cationic polyelectrolyte aqueous solution and uneven diffusion of the coagulant inside the nascent polymer film upon membrane formation via NIPS. It was also revealed that the selective layer becomes more uniform and thinner when the coagulation bath temperature increases from 25 °C to 60 °C both for reference and modified membranes (Figure 8e–h).

#### 3.4.2. AFM Studies of the Membrane Selective Layer

A number of studies have shown that membrane properties such as pure water flux, pore size or molecular weight cut-off (MWCO), hydrophilic–hydrophobic balance of the selective layer, surface charge (zeta-potential of the selective layer) and surface roughness affect the membrane fouling [1,2]. The structure of the selective layer surface of the membranes prepared using a rough glass plate was studied by the AFM technique (Figure 9 and Figure 10, Table 4). Table 4 presents the root-mean-squared surface roughness (*R*_q_) and average roughness (*R*_a_) at a scan size of 5 µm × 5 µm.

It was found that the selective layer of the reference PSF membrane and modified membranes feature the nodular morphology typical for membranes prepared by NIPS (Figure 9 and Figure 10). The reference PSF60 membrane is characterized by a relatively smooth surface of the selective layer (*R*_a_ = 4.7 nm, *R*_q_ = 6.1 nm, Table 4) with less oval and round-shaped cavities with the length of the maximum axis at 150–500 nm (Figure 9a).

It was shown that when 0.05 wt.% cationic polyelectrolyte is added to the coagulation bath the number of large cavities increases, their shape changes to more elongated one, the length of maximum axis increases up to 200–900 nm, the edges of the cavities become sharper and more pronounced (Figure 9b). These changes yield the increase of surface roughness parameters up to 9.2 nm and 11.5 nm for *R*_a_ and *R*_q_, respectively (Table 4). Upon a Praestol 859 concentration increase up to 0.1 wt.% in the coagulation bath, the size of cavities was found to increase up to maximum axis length of 1 μm and the surface of the selective layer becomes more structured and ordered (Figure 9c). The surface roughness parameters R_a_ and R_q_ were revealed to increase up to 10.2 and 13.0 nm, respectively (Figure 9c, Table 4). Furthermore, it was shown that the increase of cationic polyelectrolyte concentration up to 0.3 wt.% results in the formation of smaller round-shaped cavities with sharp high ridges (Figure 9d). However, the significant change of surface roughness parameters was not observed compared with the membrane prepared using 0.1 wt.% cationic polyelectrolyte in the coagulation bath (Table 4).

Formation of the structure with higher surface roughness upon cationic polyelectrolyte addition to the coagulation bath is due to the substantial increase of coagulant viscosity and heterogeneity and decrease of “solvent–non-solvent” exchange rate. It yields the formation of bigger polymer nodules due to a decrease in the rate of phase separation which results in the formation of bigger cavities. It is worth noting that the similar trend of the increase of roughness of the selective layer upon cationic polyelectrolyte addition to the coagulation bath is observed for membranes prepared at a coagulation bath temperature of 25 °C (Figure 9a,d, Table 3 and Table 4).

##### Effect of Temperature

To study the effect of temperature on the structure of the selective layer surface PSF membranes were prepared using water and 0.1 wt.% aqueous solution of cationic polyelectrolyte as a coagulation bath at 25 °C, 40 °C and 60 °C. A rough glass plate was applied as a support for membrane preparation via NIPS (Figure 10, Table 4).

It was found that different effects are observed when coagulation bath temperature increases in the case of using distilled water and 0.1 wt.% aqueous solution of cationic polyelectrolyte as a coagulant for membrane preparation. The size of the structural elements (cavities) on the membrane surface was found to decrease upon coagulation bath temperature increase (Figure 10a–c). However, it was revealed that surface roughness parameters of the selective layer surface practically do not change when water is used as a coagulant when coagulation bath temperature increases (Table 4, Figure 10a–c). On the contrary, the increase of coagulation bath temperature yields the change of the structure of the selective layer surface when 0.1 wt.% cationic polyelectrolyte is used: the size of the cavities increases and *R*_a_ and *R*_q_ parameters increase. However, the surface roughness parameters are practically unchanged when coagulation bath temperature increases from 40 °C to 60 °C.

### 3.5. Contact Angle Measurements

**Figure 11:**
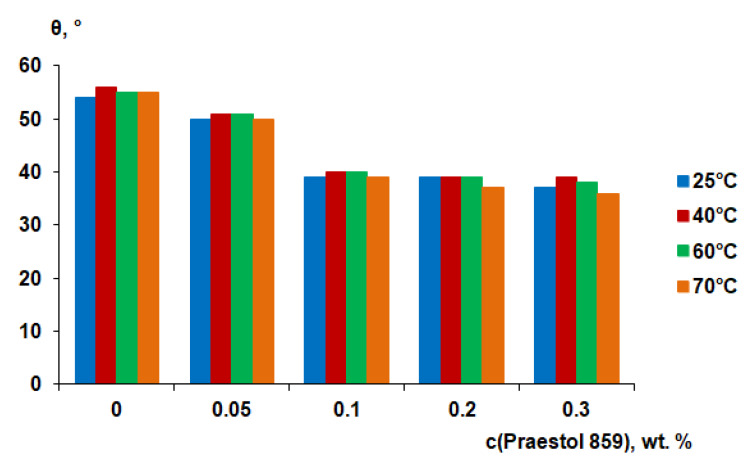
Water contact angle of the membrane selective layer of PSF-based membranes vs. Praestol 859 concentration in the coagulation bath at different temperatures.

It is generally considered that the higher the degree of hydrophilicity of the membrane surface, the better the membrane antifouling performance during ultrafiltration. It is assumed to be due to the formation of a thin layer of water on the surface of the selective layer, which prevents the adsorption of dissolved substances in the feed solution and the formation of a gel layer from them [1,2]. It was found that the water contact angle gradually decreases when cationic polyelectrolyte concentration in the coagulation bath increases (Figure 11). The water contact angle was found to be 54°–55° for reference PSF membranes, prepared at different coagulation bath temperatures. It was found that the water contact angle decreases to 50°–51° when 0.05 wt.% Praestol 859 is added to the coagulation bath (Figure 11).

It was shown that the water contact angle decreases down to 39°–40° when Praestol 859 concentration reaches 0.1 wt.% in the coagulation bath. Upon further increase of Praestol 859 concentration up to 0.2 wt.% and 0.3 wt.% the water contact angle decreases to 36°–39°. It was found that the temperature of the coagulation bath does not significantly influence the water contact angle (Figure 11). The decrease of water contact angle upon cationic polyelectrolyte addition to the coagulation bath is due to the immobilization of polyelectrolyte macromolecules into the selective layer which was confirmed by FTIR spectra (Figure 5). The hydrophilization of the selective layer is due to the presence of hydrophilic NH_2_ groups involved in hydrogen bond formation which was proven by FTIR (Figure 5). According to FTIR studies, the increase of coagulation bath temperature from 25 °C to 60 °C substantially increases the number of immobilized polyelectrolyte macromolecules which is confirmed by the increase of the intensity of the broad absorbance band in the range of 3500–3150 cm^−1^ with the maximum at 3365 cm^−1^ which corresponds to the NH_2_ groups (3 and 5 in Figure 5). However, this fact does not influence the contact angle much because the contact angle depends not only on chemical composition but also on the surface roughness parameters. It was shown that *R*_a_ and *R*_q_ increase for membranes prepared using 0.1 wt.% cationic polyelectrolyte solution as a coagulant when coagulation bath temperature increases from 25 °C to 60 °C (Table 4). The increase of surface roughness parameters counter-balances the influence of the increase of the number of hydrophilic groups on the surface of the selective layer for membranes prepared using cationic polyelectrolyte solution as a coagulation bath at 60 °C.

### 3.6. Membrane Separation Performance

For performance studies, membranes prepared using a rough glass substrate were selected due to their better membrane structure, suited for operation under pressure and leading to higher pure water flux (PWF). It was found that pure water flux of the reference PSF membrane prepared using a smooth glass plate and distilled water as a coagulant at 25 °C was 180–200 L·m^−2^·h^−1^. However, for the PSF membrane prepared using a rough glass plate and distilled water as a coagulant at 25 °C, PWF was found to be 643 L·m^−2^·h^−1^.

**Figure 12:**
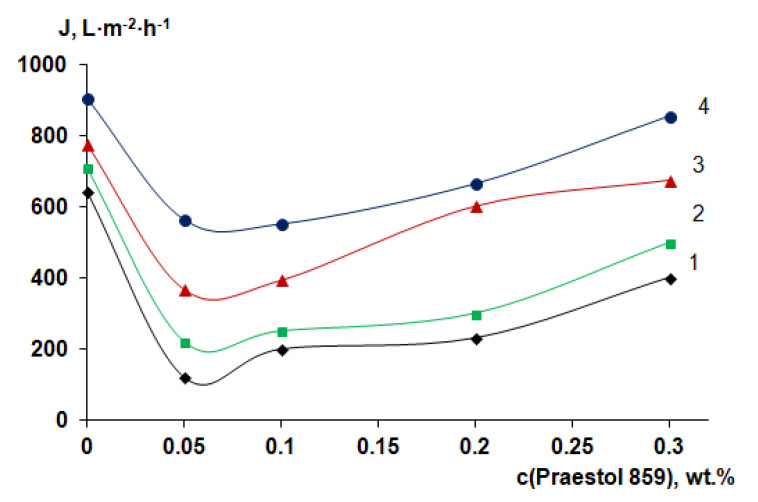
Dependence of pure water flux of PSF-based membranes on Praestol 859 concentration in the coagulation bath and coagulation bath temperature: 1—25 °C; 2—40 °C; 3—60 °C; 4—70 °C.

It was found that 0.05 wt.% cationic polyelectrolyte in the coagulation bath yields the substantial PWF decrease at all coagulation bath temperatures compared to the reference PSF membrane (Figure 12). This is due to the formation of a thicker and denser selective layer with lower porosity (Figure 7d,e). It was revealed that further increase of Praestol 859 concentration in the coagulation bath results in the increase of the PWF for all studied coagulation bath temperatures (Figure 12), which is attributed to the decrease of density and thickness of the selective layer (Figure 7e,h) and increase of selective layer hydrophilicity (Figure 11). It was shown that increase of coagulation bath temperature leads to the increase of pure water flux due to the decrease of thickness and increase of the porosity of the selective layer when coagulation bath temperature increases from 25 °C to 60 °C for both reference PSF and modified by cationic polyelectrolyte membranes (Figure 7d–f and Figure 8e–h).

**Figure 13:**
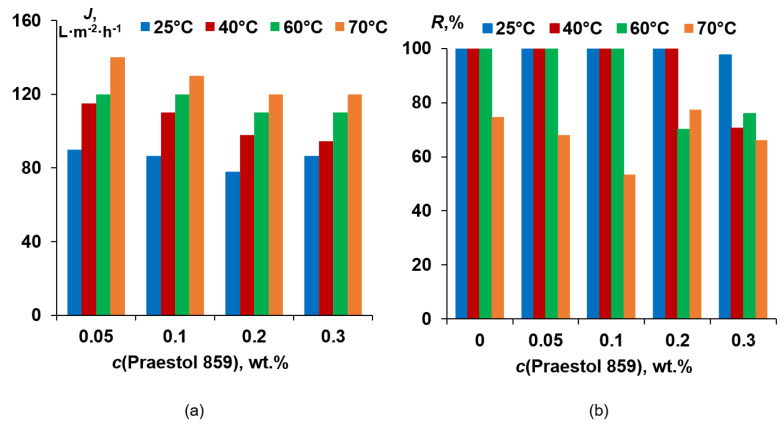
Human serum albumin (HSA) solution flux (**a**) and rejection (**b**) for PSF-based membranes vs. Praestol 859 concentration in the coagulation bath at different coagulation bath temperatures.

A flux of 0.5 wt.% HSA solution at pH = 7.0 was found to slightly decrease when Praestol 859 concentration increases in the coagulation bath (Figure 13a).

It was shown that neat PSF membranes prepared using a rough glass plate at coagulation bath temperatures of 25 °C, 40 °C and 60 °C are characterized by HSA rejection higher than 99.9% (Figure 13b). However, when the coagulation bath temperature increases up to 70 °C rejection, the coefficient was found to decrease to 74.7%. It was shown that upon cationic polyelectrolyte concentration increase up to 0.1 wt.% for coagulation bath temperatures of 25 °C, 40 °C and 60 °C and up to 0.2 wt.% for coagulation bath 25 °C and 40 °C, rejection does not change and maintain its high value (>99.9%). However, at *T* = 70 °C, rejection passes through the minimum at Praestol 859 concentration 0.1 wt.% (53.4%; Figure 13b). It was shown that for a coagulation bath temperature of 60 °C, the rejection decrease starts from 0.2 wt.% of cationic flocculant in the coagulant, and for *T* = 40 °C, rejection decrease is observed for 0.3 wt.% Praestol 859. The lowest rejection at 25 °C, 40 °C and 60 °C of coagulant is observed for 0.3 wt.% cationic flocculant in the coagulation bath (Figure 13b).

### 3.7. Evaluation of Antifouling Performance

#### 3.7.1. Human Serum Albumin Solution Ultrafiltration

To study the influence of membrane modification using cationic polyelectrolyte on membrane antifouling performance, the ultrafiltration of 0.5 wt.% HSA solutions of different pH values was carried out. PSF60 and B-0.1-60 membranes prepared using a rough glass plate were selected for HSA ultrafiltration studies. Flux recovery ratio (FRR), reversible flux decline ratio (DR_r_), irreversible flux decline ratio (DR_ir_) and total flux decline ratio (DT) were determined to evaluate membrane antifouling performance upon HSA ultrafiltration at pH 3.2, 4.6, 7.0 and 9.6. It is known that the pH of the solution and its ionic strength have a very strong effect on the change in the conformation and charge of the protein macromolecule, which is associated with the polyampholytic nature of the proteins. For HSA, the value of the isoelectric point (*pI*) is 4.6. At a pH below 2.7, the HSA is in an expanded (*E*) conformation, which is characterized by an increased size of the protein macromolecule and a significant positive charge on the surface. Between pH 2.7 and 4.3, the migrating (*F*) form is predominant, characterized by an increase in viscosity, a much lower solubility and loss of alpha helices compared to the neutral (N) form existing between pH 4.3 and 8.0. At pH above 8.0, HSA exists in the basic conformation (B), which is characterized by the loss of alpha helices. Important for this work is that at a pH below the *pI*, the HSA macromolecule has a net positive charge, and at a pH above the *pI*, it has a net negative charge. The further from the *pI* value the pH value is, the greater the charge on the surface of the protein. At the *pI*, the protein macromolecule features the smallest size and zero charge. If the pH of the HSA solution is different from *pI*, then the size of the macromolecule is increased [43,44,45].

It is worth noting that HSA rejection at all studied pH values was higher 99.9%, both for reference PSF and modified membrane.

The zeta-potential of the surface of the selective layer for reference membrane and membranes, prepared using different concentrations of Praestol 859 in the coagulation bath is presented in Table 5. It was found that at pH = 3.2, the zeta-potential of membrane modified by cationic polyelectrolyte has a much higher positive value compared to the reference membrane which is due to the quaternary ammonium groups of Praestol 859 immobilized to the selective layer. At pH = 4.6, the surface of the selective layer of the reference PSF membrane features a negative charge. The addition of 0.1 wt.% Praestol 859 to the coagulant yields the change of the charge of the selective layer to a lower absolute value (−20 mV). It is likely that immobilized Praestol 859 macromolecules are localized at certain regions of the selective layer surface and do not cover it completely. Therefore, positively charged polyelectrolyte regions contribute to the decrease of the negative charge of the PSF selective layer. With the further increase of pH value, the zeta-potential of the PSF60 membrane selective layer becomes more negative which is in a good accordance with the literature data [46]. However, those modified by cationic polyelectrolyte membrane feature even more negative zeta-potentials which is quite difficult to explain (Table 5). It may be due to the peculiarities of the pore structure of the selective layer.

**Figure 14:**
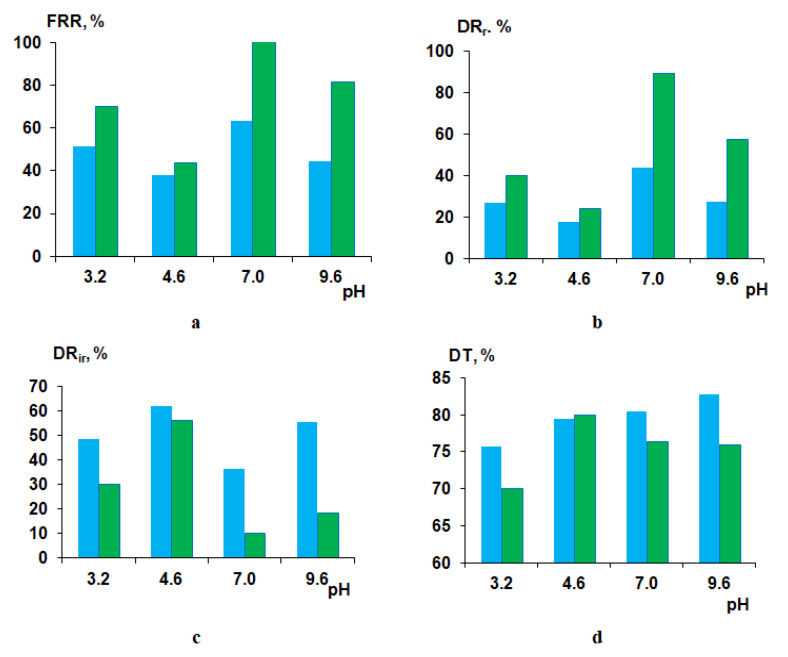
Fouling parameters of PSF60 (blue color) and B-0.1-60 (green color) membranes at different pH upon HSA solution ultrafiltration: (**a**) flux recovery ratio (FRR); (**b**) reversible flux decline ratio (DR_r_); (**c**) irreversible flux decline ratio (DR_ir_); (**d**) total flux decline ratio (DT).

It was found that at pH = 3.2, FRR for modified membrane increases up to 70% compared to the FRR of the reference unmodified membrane (51%) (Figure 14a). DR_r_ was shown to increase from 27% for the PSF60 membrane to 40% for the B-0.1-60 membrane. DR_ir_ and DT were revealed to be substantially lower for the modified membrane compared to the reference one. The enhanced antifouling performance for the B-0.1-60 membrane is due to the much higher positive zeta-potential of the selective layer which yields the electrostatic repulsion of the positively charged HSA macromolecules from the surface of the selective layer preventing them from adsorption (Table 5). Substantial higher hydrophilicity (θ = 40° for B-0.1-60 compared to θ = 55°) and lower pure water flux (392 L·m^−2^·h^−1^ for B-0.1-60 compared to 774 L·m^−2^·h^−1^ for PSF60) also contribute to the improving membrane antifouling performance despite higher surface roughness parameters compared to the reference PSF membrane (Table 4).

At pH = 4.6 FRR and DR_r_, parameters for modified membrane are slightly higher and DR_ir_ and DT parameters are slightly lower compared to the reference PSF membrane which is due to the practically similar negative zeta-potential of the selective layer surface (−28 mV for PSF60 and −20 mV for B-0.1-60; Figure 14). It is known that HSA macromolecules at *pI* feature zero charge and the smallest size compared to other pH values. Such a slight improvement of antifouling performance for the modified membrane despite higher hydrophilicity is due to the higher surface roughness of the selective layer. The compact size of HSA macromolecules allows their adhesion and deposition in the cavities which size was revealed to increase upon introduction of 0.1 wt.% of cationic polyelectrolyte to the coagulation bath (Figure 9a,c).

When HSA solution at pH = 7.0 and pH = 9.6 is used as a feed solution, the B-0.1-60 membrane was found to perform superior antifouling stability compared to the reference PSF membrane (Figure 14). At pH = 7.0 and pH = 9.6, the modified membrane was found to perform significantly higher FRR (100% and 81%, respectively), compared to the PSF60 membrane (63.4% and 44.3%), higher DR_r_ (89.2% and 57.4%, respectively) compared to the PSF60 membrane (43.9% and 27.1%, respectively) and much lower DR_ir_ and DT (Figure 14). The substantial improvement of the antifouling performance of the modified by cationic polyelectrolyte membrane is mainly attributed to the higher negative zeta-potential of the selective layer surface and higher hydrophilicity of the selective layer of modified membrane compared to unmodified one (Table 5, Figure 11).

#### 3.7.2. Surface Water Ultrafiltration

**Figure 15:**
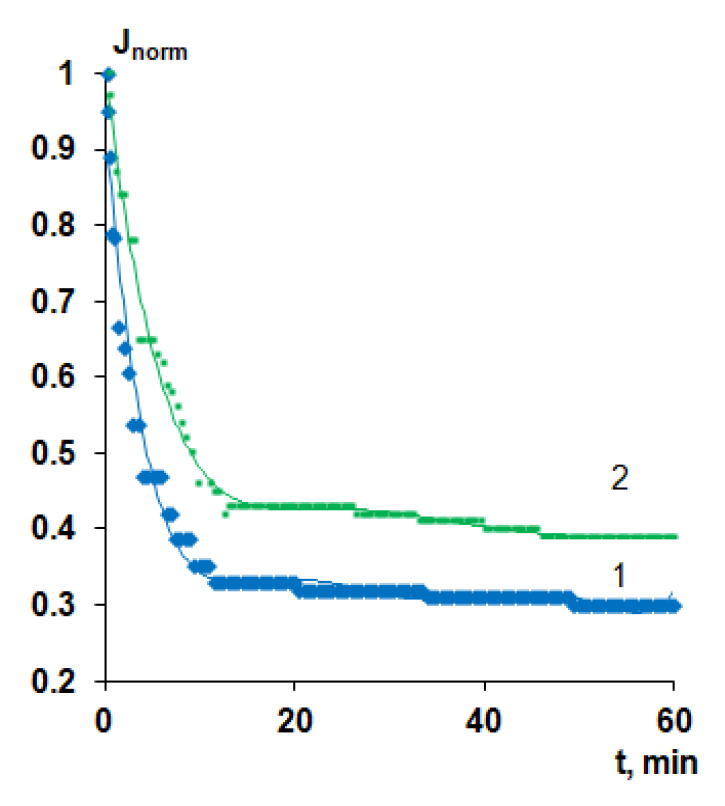
Time-dependent normalized flux (*J*/*J*_0_) of surface water (Slepianski Channel, Minsk, Belarus) during ultrafiltration of reference and modified PSF-based membranes: 1—PSF-60, 2—B-0.1-60.

Modification of polymer membranes via the addition of Praestol 859 to the coagulation bath was found to be an effective approach to increase membrane antifouling stability toward natural organic matter in the process of the filtration of surface water. Natural organic matter (NOM) consists of humic acids, fulvic acids, carbohydrates, proteins and lipids. The pH of the feed surface water was found to be 7.4 (Table 6). It is known that fulvic and humic acids which are the main constituents of the NOM feature negative charge at pH = 7.4 [47]. PSF60 and B-0.1-60 membranes were selected for surface water ultrafiltration studies. The surface water flux values at the beginning of ultrafiltration were found to be 560 L·m^−2^·h^−1^ for PSF60 and 320 L·m^−2^·h^−1^ for B-0.1-60. The substantial decrease of flux upon surface water ultrafiltration was observed both for reference and modified membrane. However, the final flux decline was found to be lower for modified by cationic polyelectrolyte membrane compared to the reference one (Figure 15). A comparison of the normalized flux of surface water allows concluding that the membrane modified using cationic polyelectrolyte is more stable to fouling by NOM compared to the reference membrane due to higher negative zeta-potential and a more hydrophilic surface of the selective layer (Figure 15, Table 5). Due to the higher negative zeta-potential of the selective layer on the modified membrane, the negatively charged fulvic and humic acids are electrostatically repelled from the membrane surface which decreases their adsorption.

It was revealed that the modified membrane more efficiently removes iron and organic carbon from the surface water (Table 6) and decreases turbidity compared to the reference PSF membrane. Improvement of rejection characteristics upon modification by cationic polyelectrolyte is due to the higher negative charge of the surface of the selective layer compared to the reference PSF membrane under the ultrafiltration conditions (Table 5 and Table 6).

In the case of NOM higher negative charge leads to the efficient electrostatic repulsion of the negatively charged fulvic and humic acids. However, in the case of iron, the electrostatic adsorption of positively charged iron ions on the negatively charged surface of the selective layer of modified membrane is responsible for the increase of the rejection and higher iron removal degree compared to the reference PSF membrane which improves the permeate quality.

## 4. Conclusions

Commercial cationic flocculant Praestol 859, a copolymer of acrylamide and 2-acryloxyethyltrimethylammonium chloride, was used for polysulfone membrane modification. The effect of the cationic polyelectrolyte addition to the coagulant on the structure, performance and antifouling stability of polysulfone membranes was studied at different coagulation bath temperatures. The immobilization of cationic polyelectrolyte macromolecules into the selective layer was confirmed by FTIR spectroscopy. It was revealed that polyelectrolyte macromolecules predominately immobilize on the surface of the selective layer but not on the bottom layer. Membrane modification was found to improve the hydrophilicity of the selective layer, to increase surface roughness and to change zeta-potential. Furthermore, it was demonstrated that the membrane barrier structure (roughness, pore size) and separation performance (permeability, rejection) can also be tuned by the polyelectrolyte concentration and temperature in the coagulation bath. Overall, the easy to perform one-step modification of polysulfone membranes via the addition of Praestol 859 to the coagulation bath in an otherwise unchanged membrane fabrication process via film casting and NIPS was found to be an effective approach to increase membrane antifouling stability toward organic fouling. Considering the polycationic structure of the surface modifier, it may also have benefits to improve biofouling resistance.

## Figures and Tables

**Table 1:** Conditions for membrane preparation.

Membrane Abbreviation	*c* (Praestol 859), wt.%	Coagulation Bath Temperature, °C
PSF25	0	25
PSF40	0	40
PSF60	0	60
B-0.05-25	0.05	25
B-0.05-40		40
B-0.05-60		60
B-0.05-70		70
B-0.1-25	0.1	25
B-0.1-40		40
B-0.1-60		60
B-0.1-70		70
B-0.2-25	0.2	25
B-0.2-40		40
B-0.2-60		60
B-0.2-70		70
B-0.3-25	0.3	25
B-0.3-40		40
B-0.3-60		60
B-0.3-70		70

**Table 2:** Composition of buffer solutions.

pH	Composition of Buffer Solution
3.0	Glycine–hydrochloric acid
4.6	Acetic acid–sodium acetate
7.0	Sodium monobasic phosphate–sodium dibasic phosphate (K_2_HPO_4_–KH_2_PO_4_)
9.6	Glycine–sodium hydroxide

**Table 3:** Membrane formation time for polysulfone (PSF) membranes at different Praestol 859 concentrations and types of glass plates used in non-solvent induced phase separation (NIPS).

Coagulation Bath		Type of Glass Support	Membrane Formation Time, s
*c* (Praestol 859), wt.%	*T*, °C		
0	25	Smooth	3
		Rough	15
0.05	25	Smooth	7
		Rough	30
0.1	25	Smooth	10
		Rough	120 ± 11
0.2	25	Smooth	100 ± 9
		Rough	300 ± 17
0.3	25	Smooth	120 ± 10
		Rough	600 ± 10

**Table 4:** Surface roughness parameters of reference PSF membranes and PSF membranes, prepared using Praestol 859 aqueous solution as a coagulant.

Membrane Abbreviation	Coagulation Bath		*R*_a_, nm	*R*_q_, nm
	*c* (Praestol 859), wt.%	T, °C		
PSF25	0	25	4.6	5.7
PSF40	0	40	4.7	5.9
PSF60	0	60	4.7	6.1
B-0.05-60	0.05	60	9.2	11.5
B-0.1-25	0.1	25	7.1	9.3
B-0.1-40		40	9.8	12.2
B-0.1-60		60	10.2	13.0
B-0.3-60	0.3	60	10.5	13.1

**Table 5:** Zeta-potential of the selective layer of reference PSF and PSF membranes, prepared using Praestol 859 aqueous solutions as coagulants.

*c* (Praestol 859), wt.%	Zeta-Potential, mV			
	pH = 3.2	pH = 4.6	pH = 7.0	pH = 9.5
0	8	−28	−58	−65
0.1	45	−20	−78	−80

**Table 6:** Characteristics of feed surface water and permeate using reference (PSF60) and modified membrane (B-0.1-60).

Characteristics	Feed Surface Water	Permeate	
		PSF60	B-0.1-60
Turbidity, NTU	12.0	0.150	0.106
Color (λ = 400 nm)	128	17	17
Total organic carbon (TOC), mg∙L^−1^	20.39	7.12	4.57
pH	7.4	7.3	7.2
c (Fe), µg∙L^−1^	410	0.70	0
